# Supplementary figures and images for: Serum Metabolomics Uncovers Immune and Lipid Pathway Alterations in Lambs Supplemented with Novel LAB-Bifidobacterium Cocktail
Source: Int J Mol Sci. 2025 Oct 9;26(19):9808. doi: 10.3390/ijms26199808 (PMC12524362; doi:10.3390/ijms26199808)

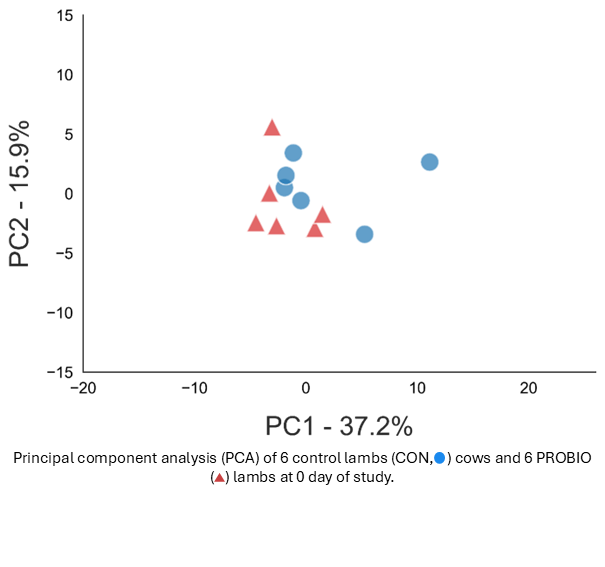

Supplement: Supplementary file 1 [file ijms-26-09808-s001.zip › ijms-3899887-supplementary.tif]
